# Oridonin attenuates the progression of atherosclerosis by inhibiting NLRP3 and activating Nrf2 in apolipoprotein E-deficient mice

**DOI:** 10.1007/s10787-023-01161-9

**Published:** 2023-05-08

**Authors:** Lei Wang, Xiaoqi Zhao, Jiawen Ding, Yutong Liu, Han Liu, Lei Zheng, Hongting Zhao, Zichen Sun, Kuanyu Li, Jing Cai, Tong Qiao

**Affiliations:** 1 Department of Vascular Surgery, Nanjing Drum Tower Hospital, Affiliated Hospital of Medical School, Nanjing University, Nanjing, 210008 Jiangsu People’s Republic of China; 2 Jiangsu Key Laboratory of Molecular Medicine, Medical School, Nanjing University, Nanjing, 210093 People’s Republic of China

**Keywords:** Atherosclerosis, Oridonin, Inflammation, Oxidative stress, Macrophages

## Abstract

Oridonin, a well-known traditional Chinese herbal medicinal product isolated from *Isodon rubescens* (Hemsl.) H.Hara, has many potential properties, including anti-inflammatory and antioxidant activities. However, there is no evidence whether oridonin have a protective effect on atherosclerosis. This study focused on the effects of oridonin on oxidative stress and inflammation generated from atherosclerosis. The therapeutic effect on atherosclerosis was evaluated by intraperitoneal injection of oridonin in a high-fat fed *ApoE*^*−/−*^ mouse model. We isolated mouse peritoneal macrophages and detected the effect of oridonin on oxidized low-density lipoprotein-induced lipid deposition. Oil red O staining, Masson's staining, dihydroethidium fluorescence staining, immunohistochemical staining, western blotting analysis, immunofluorescence, enzyme-linked immunosorbent assay and quantitative real-time PCR were used to evaluate the effect on atherosclerosis and explore the mechanisms. Oridonin treatment significantly alleviated the progression of atherosclerosis, reduced macrophage infiltration and stabilized plaques. Oridonin could significantly inhibit inflammation associated with NLRP3 activation. Oridonin significantly reduced oxidative stress by blocking Nrf2 ubiquitination and degradation. We also found that oridonin could prevent the formation of foam cells by increasing lipid efflux protein and reducing lipid uptake protein in macrophages. Oridonin has a protective effect on atherosclerosis in *ApoE*^*−/−*^ mice, which may be related to the inhibition of NLRP3 and the stabilization of Nrf2. Therefore, oridonin may be a potential therapeutic agent for atherosclerosis.

## Introduction

Atherosclerosis is the primary cause of the majority of cardiovascular diseases (CVDs), which account for more than half of all deaths worldwide (Roth et al. 2020). In recent years, great progress has been made in the scientific understanding and clinical treatment of atherosclerosis. Traditional treatments focus on reducing LDL (low-density lipoprotein) levels, but many patients still have recurrent clinical events even when they reach the target LDL level (Aday and Ridker 2019). This persistent cardiovascular risk after the best drug treatment is called residual risk. Clinical studies have now confirmed that the increased risk is mainly due to inflammation (Soehnlein and Libby 2021).

Therefore, it is proposed that pharmacological targeting of inflammatory pathways might improve the outcomes of cardiovascular outcomes in patients with cardiovascular diseases (Grebe et al. 2018). IL (interleukin)-1 family cytokines are major mediators of vascular and systemic inflammation, which promote the formation of atherosclerosis (Galea et al. 1996). The NLRP3 [NOD (nucleotide oligomerization domain)-, LRR (leucine-rich repeat)-, and PYD (pyrin domain)-containing protein 3] inflammasome is the key mediator of IL-1 family cytokine production in atherosclerosis (Grebe et al. 2018). NLRP3 inflammasome is constituted of the proteins ASC (apoptosis-associated speck-like protein containing a C terminal caspase recruitment domain), caspase-1 and NLRP3. NLRP3 is activated by a variety of endogenous danger signals, such as oxidized low-density lipoprotein (ox-LDL) and cholesterol crystals, which are abundant in macrophages of atherosclerotic lesions. (Liu et al. 2014; Shao et al. 2022). Once stimulated, caspase-1 is activated by the NLRP3 inflammasome, resulting in the maturation of pro-IL-1β from an inactive state. Increasing evidence suggests that the NLRP3 inflammasome plays an essential role in atherosclerotic development (Shao et al. 2022). Previous studies have revealed that the activation of NLRP3 inflammasome contributes to the vascular inflammatory response, which leads to cholesterol accumulation by impairing cellular cholesterol efflux (Tall and Yvan-Charvet 2015). Emerging evidences indicate that suppressing NLRP3 inflammasome activation alleviates atherosclerotic progression (Ma et al. 2018; Zhao et al. 2020; Zheng et al. 2014).

Oxidative stress, which also happens in atherosclerosis, occurs when the continual generation of reactive oxygen species (ROS) overloads the capabilities of the organic antioxidative defense system and causes damage to DNA, proteins, and lipids (Wang and Bennett 2012). The inflammatory factors such as IL-1β which are activated by the NLRP3 inflammasome in atherosclerosis activate polymorphonuclear neutrophils, inducing “respiratory burst” and producing a large amount of ROS (Gross et al. 2011; Martinon et al. 2002). ROS can increase the expression of ox-LDL receptors on the cell surface, such as a cluster of differentiation 36 (CD36), which play critical roles in the formation of foam cells by increasing ox-LDL influx (Fuhrman et al. 2002). Multiple pathways of the cellular anti-oxidative stress response are regulated by the “master regulator”, Nuclear factor erythroid 2-related factor 2 (Nrf2) (Hybertson et al. 2011). As a vital nuclear transcriptional factor, Nrf2 shows strong antioxidative activity and has been widely used as a promoter to suppress oxidative stress (Hybertson et al. 2011). Normally, Nrf2 interacts with Kelch-like epichlorohydrin-related proteins (Keap1) to form low-activity complexes in the cytoplasm (Li and Kong 2009). In response to oxidative stress, Nrf2 is released from the complex and enters the nucleus to regulate the expression of genes containing antioxidant response elements (AREs), such as heme oxygenase (HO-1) (Tkachev et al. 2011).

Numerous compounds interact with the NLRP3 inflammasome and Nrf2 to provide anti-inflammatory and antioxidative properties. The natural compound oridonin, derived from the plant *Rabdosia Rrubescens*, has been studied as a covalent NLRP3 inhibitor (He et al. 2018). Furthermore, it was demonstrated that oridonin exerted protective effects on LPS-induced acute lung injury and hind limb ischemia–reperfusion injury via Nrf2-independent anti-inflammatory and Nrf2-dependent antioxidative activities (Yang et al. 2019; Zhao et al. 2022). However, there is no evidence whether oridonin have a protective effect on atherosclerosis. Thus, in the present study, we focused on the protective effects of oridonin on inflammation and oxidative stress generated from atherosclerosis.

## Materials and methods

### Animals

Apolipoprotein E-deficient (*ApoE*^*−/−*^) mice (8 weeks old), the most popular murine model for atherosclerotic study, were used in this study. *ApoE*^*−/−*^ mice were purchased from the Model Animal Research Center of Nanjing University (Nanjing, China). At the age of eight weeks, male *ApoE*^*−/−*^ mice were fed a high-fat diet containing 0.2% cholesterol and 20% fat for 12 weeks in an SPF animal facility with free access to water. The mice were randomly divided into three groups: atherosclerosis (AS) group and two AS + oridonin groups (10 and 20 mg/kg/day). The body weight and food intake of mice in all groups were monitored weekly throughout the treatment period. All protocols were approved by the Animal Investigation Ethics Committee of The Affiliated Drum Tower Hospital of Nanjing University Medical School and were performed according to the Guidelines for the Care and Use of Laboratory Animals published by the National Institutes of Health, USA.

### Drug administration

Oridonin (purity > 99.81%) was purchased from Selleck Chemicals (Shanghai; China). Supplementary Fig. S1 presents the high-performance liquid chromatography (HPLC) of oridonin. A solution of 1% dimethyl sulfoxide (DMSO) with normal saline was used to dissolve oridonin. The dose of oridonin (10 mg/kg/day and 20 mg/kg/day) was chosen based on previous studies (Lu et al. 2020; Yan et al. 2020; Zhao et al. 2022). The mice were intraperitoneally injected with normal saline or oridonin (10 mg/kg or 20 mg/kg) daily for 12 weeks from 8 weeks of age.

### Blood and tissue collection

All animals were anesthetized with an intraperitoneal injection of pentobarbital sodium (40 mg/kg) and euthanized by cervical dislocation. In order to obtain serum, blood samples were collected and centrifuged 2500×*g* at 4 °C for 15 min. Serum samples were then stored at – 80 °C for the determination of serum IL-6, CRP, cholesterol and triglycerides. After perfusion, the arteries and heart were collected and soaked in 4% paraformaldehyde for histological analysis or quickly frozen at – 80 °C for further analysis. All experimental procedures involving laboratory animals were approved by the Animal Investigation Ethics Committee of The Affiliated Drum Tower Hospital of Nanjing University Medical School.

### Atherosclerotic plaque assessment

Each aorta is separated from the aortic arch to the bifurcation of the iliac artery. after removing the adventitia connective tissue, the aorta was cut longitudinally and laid on black wax paper. For analysis of the intraluminal lesions area, Oil Red O staining was performed. For analysis of atherosclerotic lesion size in the aortic sinus, the proximal aorta attached to the heart was embedded in paraffin or frozen in the Tissue-Tek OCT compound. Serial 6-μm-thick sections of the aortic sinus with valves were collected and stained with Masson's trichrome and oil red O staining. Additional sections were used for immunohistochemical/immunofluorescence staining. The quantification of atherosclerotic lesions area and size were analyzed using Image J software after staining by two observers who were blind to the experimental groups allocation.

### Isolation of peritoneal macrophages

Peritoneal macrophages were isolated as follows. Mice were injected intraperitoneally with 1 mL 4% BBL thioglycollate, Brewer modified (BD Biosciences, Shanghai, China). After 3 days, primary peritoneal macrophages were collected from euthanized animals using 8 mL cold PBS. Then, the cells were cultured for 8 h in RPMI 1640 medium supplemented with 10% fetal bovine serum. The macrophages were subsequently cultured for 48 h in a medium containing 50 μg/mL human oxidized low-density lipoprotein (ox-LDL) with the treatment of 2.5 and 5 μM oridonin. Oil Red O staining was used to assess foam cell formation.

### Statistical analysis

The results are presented as means ± SEM. Differences between mean values of normally distributed data were analyzed using one-way ANOVA. GraphPad Prism software 8.0 was used for all analyses. Statistical significance was considered when *p* < 0.05.

## Results

### Oridonin inhibits the progression of atherosclerosis in ***ApoE***^***−/−***^ mice

**Fig. 1:**
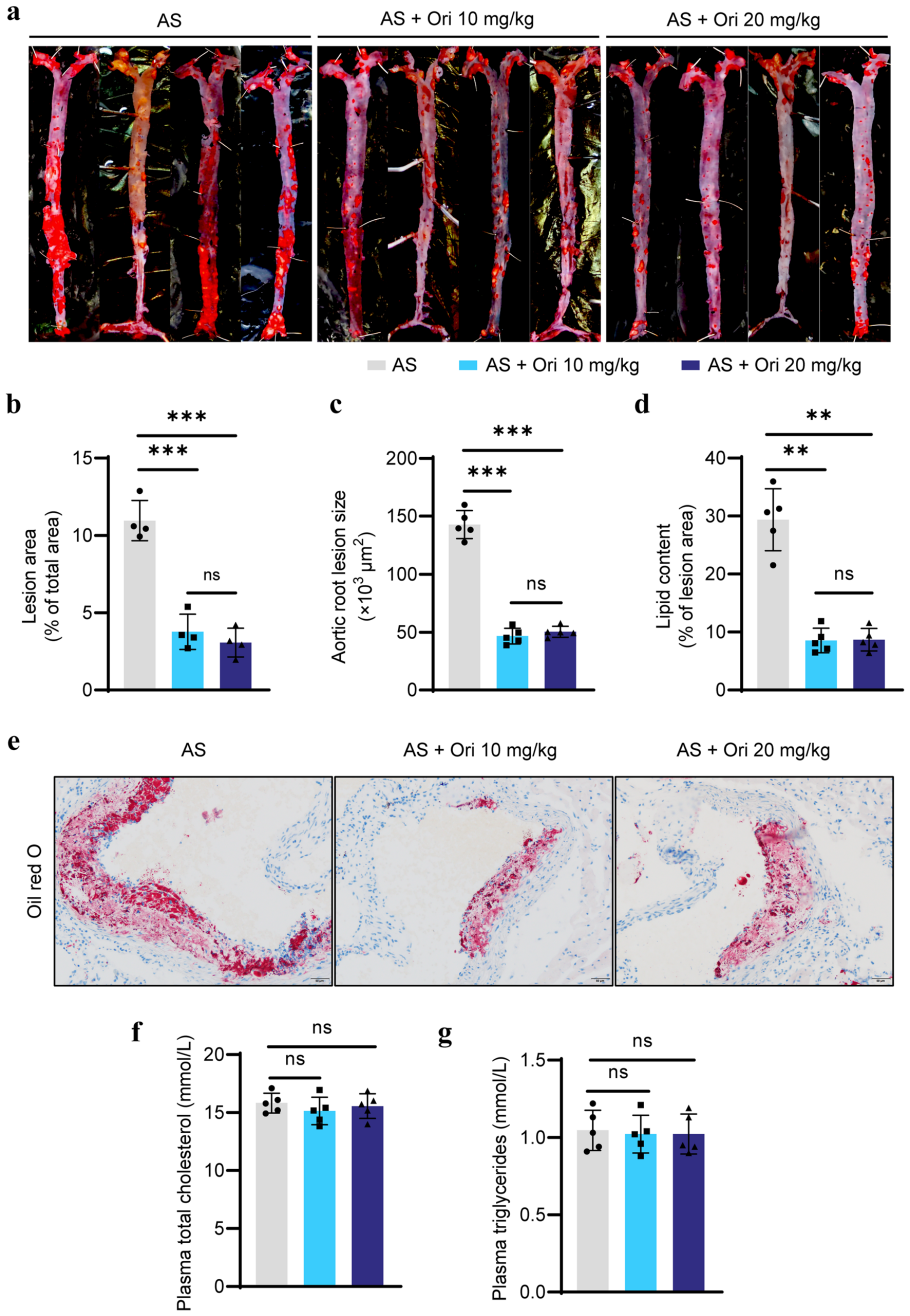
Oridonin ameliorated atherosclerotic progression in *ApoE*^*−/−*^ mice. High-fat-fed *ApoE*^*−/−*^ mice received daily intraperitoneal injections of 10 mg/kg oridonin or 20 mg/kg oridonin, or saline for 12 weeks. **a** The representative photographs of oil Red O staining of lumen surface of aortas in *ApoE*^*−/−*^ mice with different treatments as indicated. **b** Quantification of the lesion area for **a**, *n* = 4. **e** The oil Red O staining of aortic root sections. **c** Quantification of the aortic root lesion size for **e**, *n* = 5. **d** Quantification of lipid content in aortic root lesions for **e**, *n* = 5. **f, g** Plasma cholesterol and triglyceride in different groups, *n* = 5. Data are presented as the mean ± SEM. Statistical significance was determined using one way ANOVA. *p < 0.05, **p < 0.01, ***p < 0.001, ns, no significance

To explore the possible effect of oridonin on the progression of atherosclerosis in vivo, *ApoE*^*−/−*^ mice fed with a high-fat diet were intraperitoneally injected with 10 mg/kg (AS + Ori 10 mg/kg group) or 20 mg/kg oridonin (AS + Ori 20 mg/kg group) or normal saline (AS group) every day for 12 weeks. The formation of atherosclerotic lesions was evaluated by oil red O staining in the aortas. The area of the oil red positive lesion was dramatically reduced in the treatment groups compared to the AS group (Fig. 1a, b). The oil red O staining area of the aortic sinus in the treatment groups was less than that in the AS group, which was consistent with the decrease in overall lesion area (Fig. 1d, e). In accordance with the decrease in lipid content, aortic root lesion size was also decreased in treatment groups (Fig. 1c). In addition, oridonin had no significant effect on overall body weight (data not shown), the levels of circulating total cholesterol (TC) (Fig. 1f) and total triglyceride (TG) (Fig. 1g) in *ApoE*^*−/−*^ mice.

**Fig. 2:**
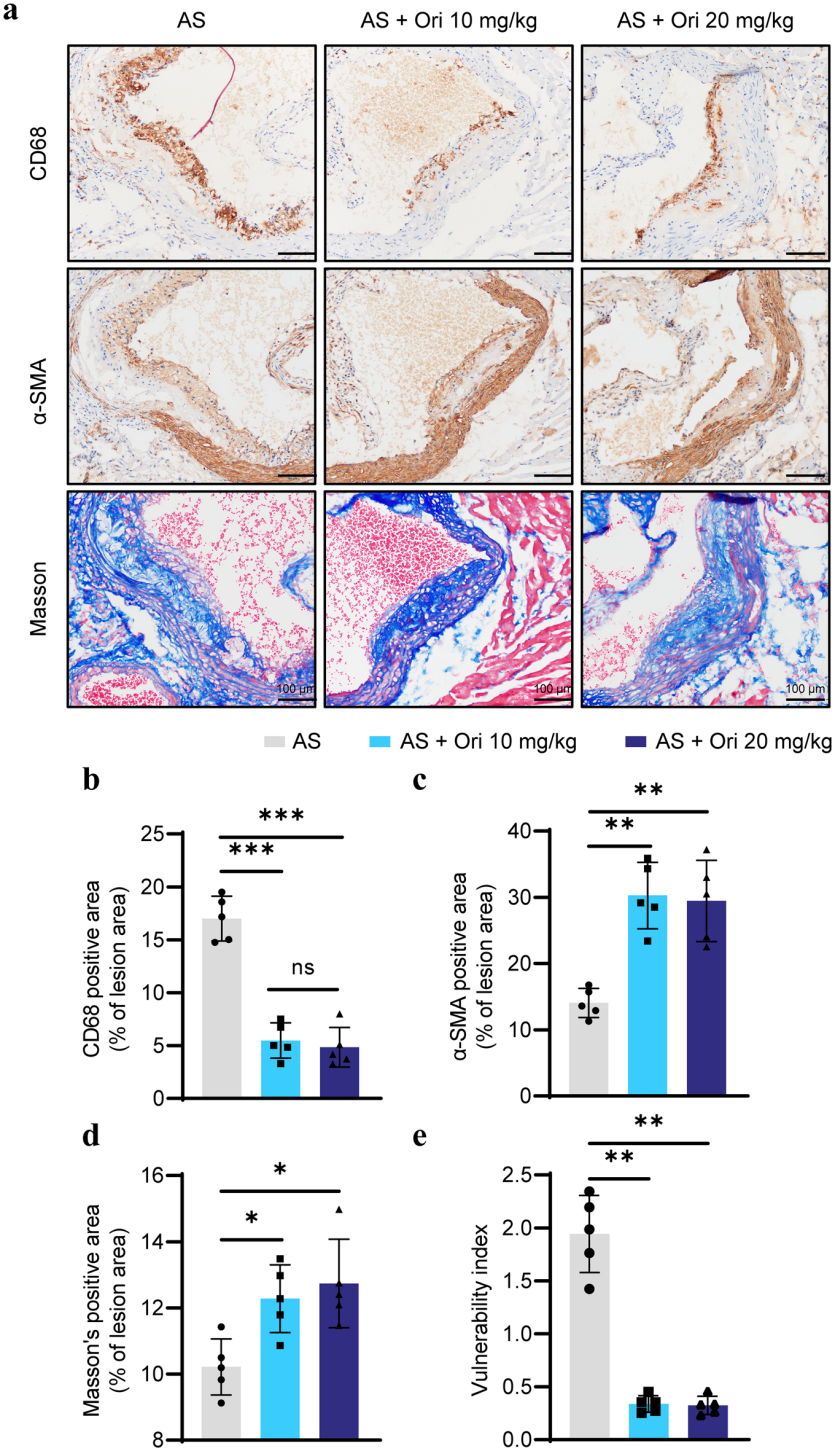
Oridonin reduced macrophage infiltration in plaque and increased the stability of atherosclerotic plaque. **a** Representative images of immunohistochemical staining for CD68 (as a marker of macrophages), α-SMA (as a marker of smooth muscle), and Masson staining for collagenous fibers in aortic root lesions. The quantification of stained areas is presented as the mean ± SEM in **b**–**d**, *n* = 5. **e** The vulnerability index was calculated by dividing the sum of the plaque area stained positive for CD68, and Oil red O staining with the plaque area stained positive for α-SMA and collagen. Statistical significance was determined using one way ANOVA. *p < 0.05, **p < 0.01, and ***p < 0.001, ns, no significance

To further study the plaque, the aortic sinus sections of mice were stained with immunohistochemistry to evaluate its composition. CD68 (a marker of infiltration macrophages), α-SMA (a marker of vascular smooth muscle cells), and Masson staining (the level of collagen) were detected in the aortic sinus sections. As anticipated, oridonin treatment significantly decreased the number of infiltrated macrophages (Fig. 2a, b). Additionally, a significant increase of α-SMA and Masson staining positive area was observed in the atherosclerotic plaque sections (Fig. 2a, c, d). Plaque vulnerability index showed that oridonin treatment made plaque more stable (Fig. 2e). The vulnerability index was calculated by dividing the sum of the plaque area stained positive for CD68, and Oil red O staining with the plaque area stained positive for α-SMA and collagen (Edsfeldt et al. 2022). Taken together, these results revealed that the mice in the oridonin treatment groups had a lower lesion area than mice in AS group, and the plaque was more stable.

### Oridonin reduces systemic and arterial inflammation by inhibiting NLRP3 inflammasome in *ApoE*^*−/−*^ mice

**Fig. 3:**
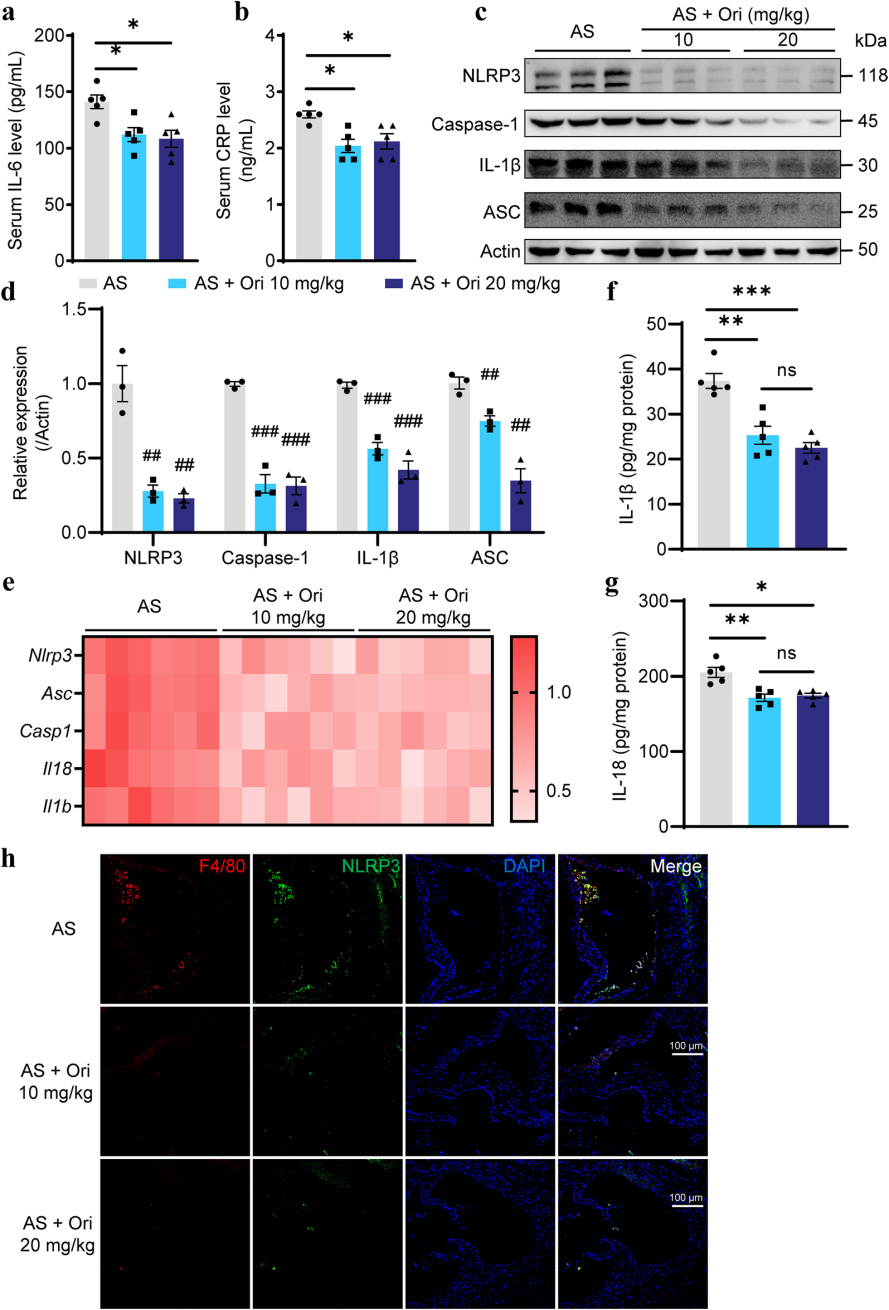
Oridonin reduced the level of systemic inflammation and the activation of NLRP3 in aortic tissue. Determination of the concentrations of serum IL-6 (**a**) and serum CRP (**b**), *n* = 5. **c** Western blot analysis of NLRP3, Caspase-1, IL-1β, and ASC in the aortas of *ApoE*^*−/−*^ mice with different treatments. **d** Relative protein levels of NLRP3, Caspase-1, IL-1β, and ASC from western blot were quantified. **e** Relative mRNA levels of *Nlrp3*, *Asc*, *Casp1*, *Il18* and *Il1b* were detected by qPCR, *n* = 6. IL-1β (**f**) and IL-18 (**g**) levels were measured by the ELISA assay within aortas, *n* = 5. **h** Immunofluorescence colocalization of F4/80 and NLRP3 in aortic sinus of *ApoE*^*−/−*^ mice with different treatments. Scale bar = 100 μm. Data were shown as mean ± SEM. Statistical significance was determined using one way ANOVA. *p < 0.05, **p < 0.01, and ***p < 0.001, ns, no significance. ^#^*p* < 0.05, ^##^*p* < 0.01, ^###^*p* < 0.001 compared with the AS group

Because oridonin is considered to be an effective NLRP3 inhibitor (He et al. 2018), we investigated whether it can reduce inflammation in *ApoE*^*−/−*^ mice. Administration of low-dose (10 mg/kg) and high-dose (20 mg/kg) oridonin significantly decreased serum IL-6 and CRP levels (Fig. 3a, b). It suggested that oridonin reduced systemic inflammation levels in *ApoE*^*−/−*^ mice. Subsequently, we assessed whether local inflammation levels and the NLRP3 pathway were down-regulated in oridonin-treated aortic tissues. Significantly decreased protein and mRNA levels of NLRP3, Caspase-1, IL-1β and ASC were detected in the arteries of treatment groups (Fig. 3c–e). The IL-1β and IL-18 levels in arteries were detected by ELISA and were found to be decreased in oridonin treatment groups (Fig. 3f, g). In addition, immunofluorescence showed that the co-localization of F4/80 (the marker of macrophages) and NLRP3 was significantly decreased in oridonin treatment groups (Fig. 3h). Consistent with CD68 immunohistochemical staining, it showed that oridonin reduced macrophage infiltration and suppressed NLRP3 expression. Collectively, oridonin administration decreased inflammatory cytokines production and NLRP3 activation in the arteries of mice. The level of systemic inflammation also decreased in oridonin treatment groups.

### Oridonin reduces oxidative stress by activating Nrf2 pathway in aortic plaques of *ApoE*^*−/−*^ mice

**Fig. 4:**
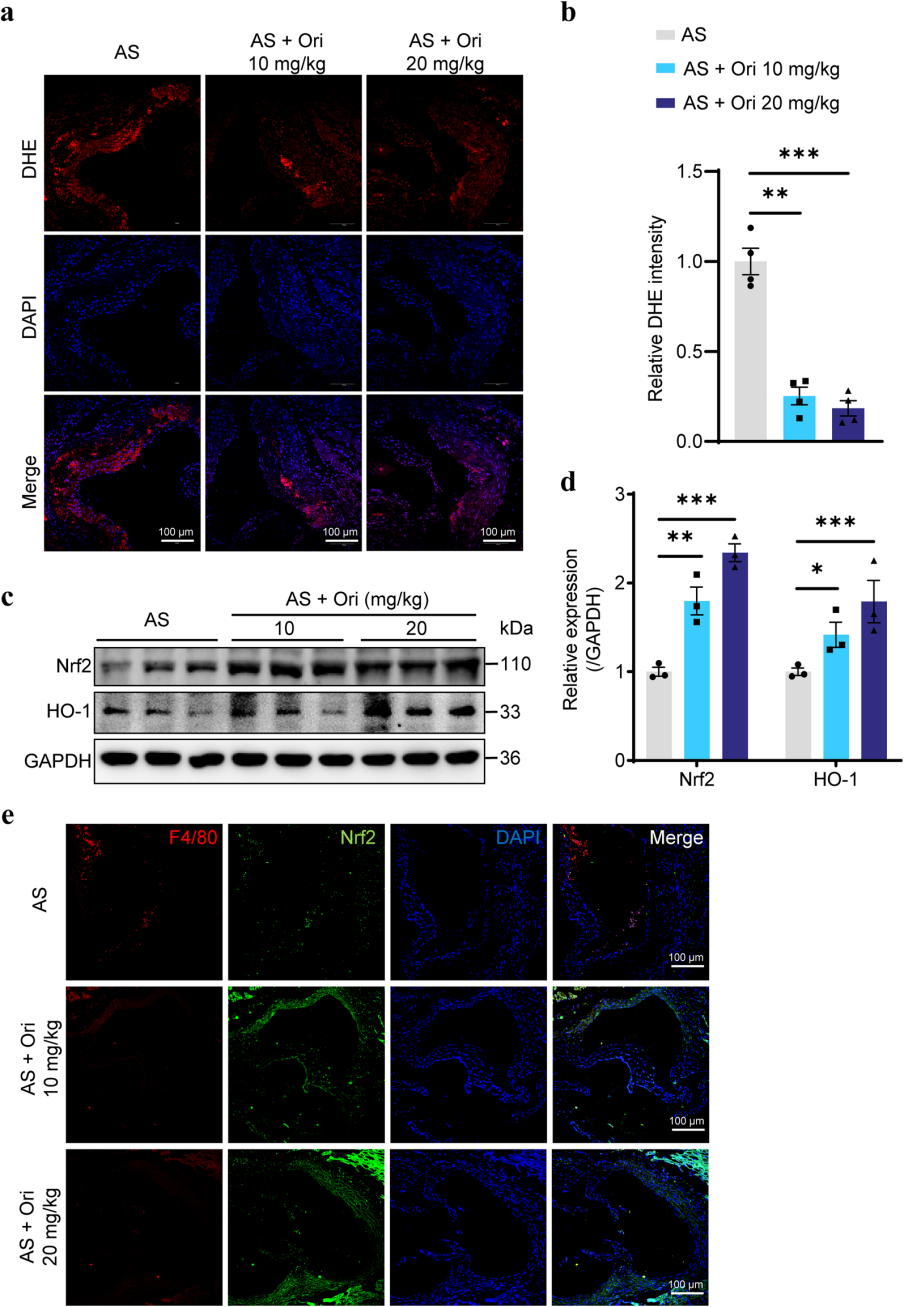
Oridonin reduced the accumulation of ROS in aortic root lesions and enhanced the ability of antioxidant stress in aortic tissue. **a** Representative images of DHE staining in the aortic sinus and quantification of DHE fluorescence intensity (**b**), *n* = 4. **c** Western blot analysis of Nrf2 and HO-1 in the aortas of different groups. **d** Relative protein levels were quantified for **c**. **e** Immunofluorescence colocalization of F4/80 and Nrf2 in aortic sinus of *ApoE*^*−/−*^ mice with different treatments. Scale bar = 100 μm. Data were shown as mean ± SEM. Statistical significance was determined using one way ANOVA. *p < 0.05, **p < 0.01, and ***p < 0.001, ns, no significance

As previously mentioned, inflammatory factors in atherosclerotic plaques lead to the accumulation of ROS, triggering oxidative stress, which is also closely related to the progression of atherosclerosis. Oridonin has been investigated as an Nrf2 activator with antioxidative activities (Du et al. 2008; Yang et al. 2019; Zhao et al. 2022). Therefore, we studied whether oridonin could reduce the accumulation of ROS in the aortas of *ApoE*^*−/−*^ mice. Dihydroethidium (DHE) fluorescence staining was used to detect ROS in aortic sinus sections. The results showed that oridonin treatment reduced the level of ROS in aortic plaques (Fig. 4a, b). To further examine the anti-oxidative stress capacity in aortic tissue after oridonin treatment, we examined the protein expression of Nrf2 and HO-1. Compared with the AS group, the protein expression levels of antioxidant genes Nrf2 and HO-1 in the aortas of mice treated with oridonin were significantly increased (Fig. 4c, d). Immunofluorescence staining showed that oridonin decreased the infiltration of macrophages in the plaque while enhancing the expression of Nrf2 in the plaque (Fig. 4e). In general, oridonin improved the ability of aortic tissue to resist oxidant stress by increasing the expression of Nrf2.

### Oridonin inhibits NLRP3 activation and upregulates the Nrf2 protein level in peritoneal macrophages in vitro

**Fig. 5:**
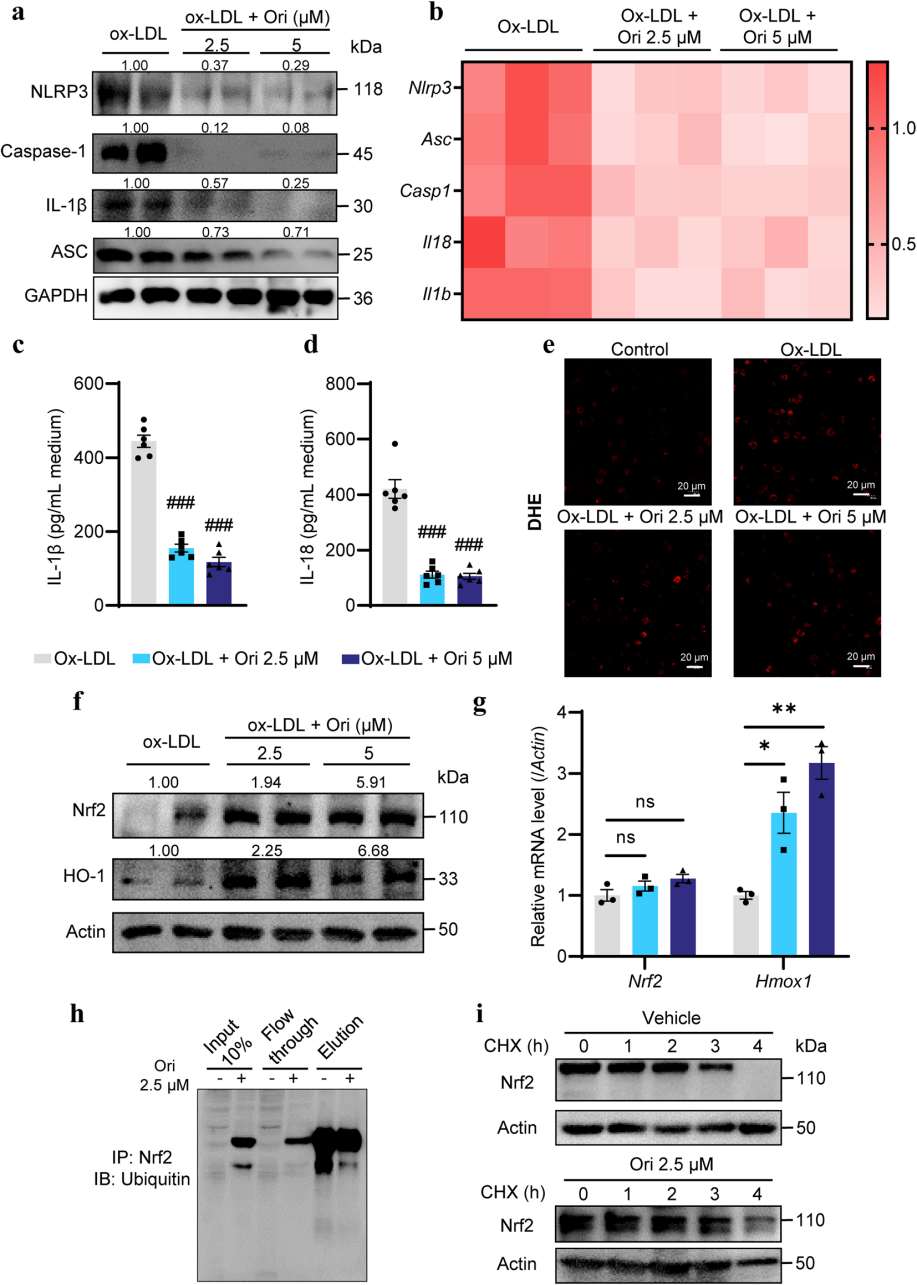
Oridonin inhibited ox-LDL induced NLRP3 inflammasome activation and enhanced the ability of antioxidant stress by up-regulation of the Nrf2 protein level in peritoneal-derived macrophages. Peritoneal-derived macrophages were exposed to ox-LDL (50 μg/mL) in the presence or absence of Oridonin (2.5 or 5 μM) for 24 h. **a** Western blot analysis of NLRP3, Caspase-1, IL-1β and ASC in peritoneal derived macrophages with different treatments as indicated. **b** Relative mRNA levels of *Nlrp3*, *Asc*, *Casp1*, *Il18* and *Il1b* were detected by qPCR, *n* = 3. IL-1β (**c**) and IL-18 (**d**) levels were measured by the ELISA assay within the medium, *n* = 6. **e** Representative images of DHE staining in peritoneal derived macrophages of different groups. Scale bar = 20 μm. **f** Western blot analysis of Nrf2 and HO-1 in peritoneal derived macrophages with different treatments as indicated. **g** Relative mRNA levels of *Nrf2* and *Hmox1* were detected by qPCR, *n* = 3. **h** Ubiquitination of Nrf2 evaluated in Raw264.7 cells treated with DMSO or 2.5 μM oridonin for 4 h, along with 10 μM MG132; Nrf2 was immunoprecipitated with anti-Nrf2 antibody, and ubiquitinated Nrf2 was detected with anti-ubiquitin antibody. **i** Protein half-life in Raw264.7 cells untreated or treated with 2.5 μM Oridonin for 4 h. Cycloheximide (50 μM) was added to block protein synthesis. Cells were lysed at the indicated time points, and lysates were subjected for immunoblot analysis of Nrf2. Data were shown as mean ± SEM. Statistical significance was determined using one way ANOVA. *p < 0.05 and **p < 0.01, ns, no significance. ^#^*p* < 0.05, ^##^*p* < 0.01, ^###^*p* < 0.001 compared with the AS group.

To further verify the effect of oridonin on the inflammatory response and antioxidant response in atherosclerotic plaque, we isolated primary peritoneal macrophages from *ApoE*^*−/−*^ mice. Then the cells were exposed to ox-LDL (50 μg/mL) for 48 h in the presence or absence of oridonin (2.5 μM and 5 μM) to mimic macrophages in plaques. To evaluate the inhibitory effect of oridonin on ox-LDL-induced inflammation, we examined the expression levels of NLRP3 pathway-related genes and the levels of cytokines in the medium. The protein and mRNA expression levels of NLRP3, Caspase-1, IL-1β and ASC were significantly decreased in oridonin treatment groups (Fig. 5a, b). After oridonin treatment, the IL-1β and IL-18 secretion levels in the medium were detected by ELISA. There was a significant difference between the treatment groups and the control group (Fig. 5c, d). Overall, these results indicate that oridonin can inhibit NLRP3 activation and inflammation cytokines release in ox-LDL induced peritoneal macrophages.

Then we evaluated the antioxidant stress ability of ox-LDL-induced peritoneal macrophages after oridonin treatment. To evaluate ROS production, we utilized DHE fluorescent staining. The macrophages with oridonin treatment showed less red fluorescence than the control group (Fig. 5e). Consistent with the protein expression levels of antioxidant genes in the aortas, oridonin treatment increased the expression of Nrf2 and HO-1 in macrophages (Fig. 5f). To explore how oridonin upregulates the protein expression of Nrf2, we first detected the transcriptional levels of *Nrf2* and *Hmox1* by qPCR. As shown in Fig. 5g, there was no statistically significant difference in *Nrf2* mRNA with treatment of oridonin. The mRNA level of *Hmox1* was induced significantly by oridonin. These data showed that oridonin can activate the Nrf2 signaling pathway primarily by increasing the Nrf2 protein level.

Previous studies have shown that Nrf2 activator induces Nrf2 signal pathway mainly by interfering with keap1-dependent ubiquitin coupling mechanism (Nguyen et al. 2003). Studies have found that oridonin can increase the expression level of Nrf2 in human breast carcinoma cells (Du et al. 2008). We thus evaluated the capacity of oridonin to modulate Nrf2 ubiquitination. Immunoprecipitation showed oridonin suppressed Nrf2 ubiquitination in Raw264.7 cells (Fig. 5h). We also measured the half-life of Nrf2 in Raw264.7 with or without oridonin treatment. Treatment with oridonin enhanced the half-life of the Nrf2 protein in Raw264.7 cells (Fig. 5i). These data revealed that oridonin increased the stability of Nrf2 by blocking Nrf2 ubiquitination in macrophages.

### Oridonin inhibits lipid uptake and enhances lipid efflux in macrophages in vivo and in vitro

Foam cell production is a critical stage in the development of atherosclerotic plaque. Uncontrolled ox-LDL absorption, inordinate cholesterol esterification and blocked cholesterol excretion result in the accumulation of cholesterol ester in the form of lipid droplets, which causes the formation of foam cells (Groenen et al. 2021). As mentioned earlier, the levels of inflammation and oxidative stress affect the lipid metabolism of macrophages. Therefore, we asked whether the inhibition of oridonin on inflammation and oxidative stress could improve the lipid-handling ability of macrophages. Previous studies have reported that selective NLRP3 inflammasome inhibitors decrease foam cell formation via suppression of ox-LDL uptake and enhancement of cholesterol efflux (Chen et al. 2018). Therefore, we speculated whether oridonin treatment could inhibit the formation of foam cells by regulating the “input” and “output” of lipids.

**Fig. 6:**
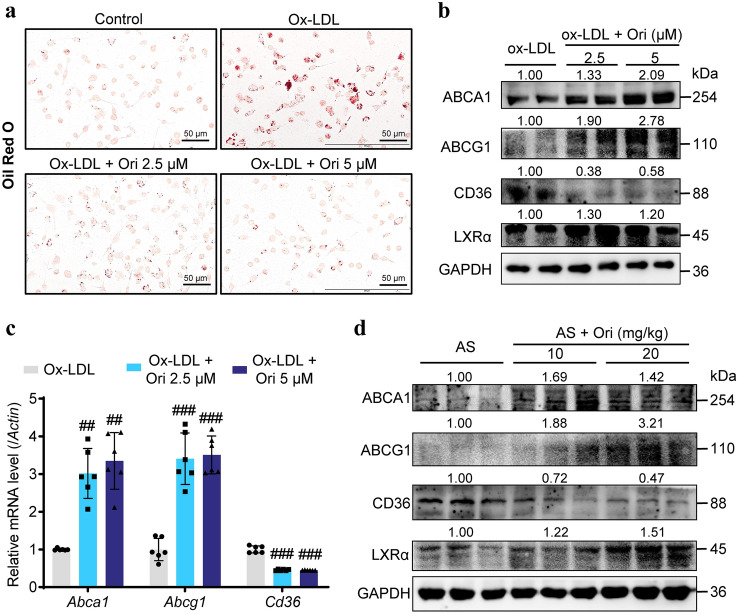
Oridonin reduced lipid accumulation in macrophages, associated with enhanced lipid efflux and decreased lipid influx. **a** Representative images of Oil Red O-stained peritoneal derived macrophages. Scale bar = 50 μm. **b** Western blot analysis of ABCA1, ABCG1, CD36 and LXRα in peritoneal derived macrophages with different treatments as indicated. **c** Western blot analysis of ABCA1, ABCG1, CD36 and LXRα in the aortas of different groups. **d** Relative mRNA levels of *Abca1*, *Abcg1* and *Cd36* in peritoneal-derived macrophages of different groups were detected by qPCR, *n* = 6. Data were shown as mean ± SEM. Statistical significance was determined using one way ANOVA. *p < 0.05, **p < 0.01, and ***p < 0.001

The primary peritoneal macrophages from *ApoE*^*−/−*^ mice were exposed to ox-LDL (50 μg/mL) for 48 h with or without oridonin. Oil red O staining showed oridonin decreased ox-LDL-induced lipid deposition in peritoneal macrophages (Fig. 6a). Then, we detected the expression of CD36, the main receptors responsible for the ox-LDL influx, ABCA1 and ABCG1 (ATP Binding Cassette A1 and G1 cholesterol transporters), two transporters mediating cholesterol efflux (Yvan-Charvet et al. 2019). Oridonin treatment group significantly down-regulated CD36 and up-regulated ABCA1 and ABCG1 in ox-LDL induced macrophages (Fig. 6b, c). The expression of lipid flow-related proteins CD36, ABCA1 and ABCG1 was also detected in aortic plaques by Western blot (Fig. 6d). According to the in vitro and in vivo data, oridonin can inhibit intracellular lipid accumulation, and its mechanism may be related to the decrease of CD36 and up-regulation of ABCA1 and ABCG1 expression, inhibition of ox-LDL uptake and promotion of lipid excretion, thus preventing the formation of foam cells.

ABCA1 and ABCG1 expression are positively regulated by the nuclear receptor liver X receptor (LXR), which forms a heterodimer by binding with the retinoid X receptor, and acts as a transcription factor (Yvan-Charvet et al. 2019). Thus, we wondered whether LXRα was upregulated to increase the expression of ABCA1 and ABCG1. As shown in Fig. 6b, d, oridonin treatment up-regulated the protein level of LXRα. These data demonstrated that oridonin alleviates lipid deposition in macrophages by upregulation of LXRα-ABCA1/ABCG1 and downregulation of CD36.

## Discussion

As early as the nineteenth century, pathologists Rokitansky and Virchow described the inflammatory characteristics of atherosclerotic plaques (Grebe et al. 2018). In recent years, multiple clinical studies have confirmed that the residual risk after traditional LDL-lowering therapy is mainly due to inflammation (Doran 2022). According to the Canakinumab Anti-inflammatory Thrombosis Outcome Study (CANTOS), lowering low-grade systemic inflammation in CVD patients reduced the risk of acute cardiovascular events without affecting blood cholesterol levels (Ridker et al. 2017). However, systemic targeting of inflammatory pathways always carries the risk of disrupting immune homeostasis and negatively impacting protective immune responses, such as during infection (Ridker et al. 2017). The CANTOS showed that canakinumab increased the risk of sepsis and deadly infections (Ridker et al. 2017). Pharmacological suppression of NLRP3 inflammasome activation may provide a more targeted and advantageous treatment strategy for CVDs (Grebe et al. 2018; Satish and Agrawal 2020; Shao et al. 2022). In comparison to canakinumab, which all block IL-1β function, the risk of fatal infections is predicted to be decreased due to specific NLRP3 inflammasome inhibition (Soehnlein and Libby 2021). Multiple studies have demonstrated that inhibition of NLRP3 activation has a protective effect on atherosclerosis (Hu et al. 2018; Shao et al. 2022; Zhao et al. 2020).

Oridonin, a diterpenoid isolated from *R. rubescens*, has anti-inflammatory and antioxidant activities (Owona and Schluesener 2015). Recent studies have revealed that oridonin can bind covalently to NLRP3, resulting in potent anti-inflammatory activity both in vivo and in vitro (He et al. 2018). Oridonin has been used to treat various diseases for a long time, and modern science gave the pharmacological effects of oridonin a solid scientific foundation. For instance, oridonin can alleviate carrageenan-induced pleurisy by activating the KEAP-1/Nrf2 pathway and inhibiting the TXNIP/NLRP3 and NF-κB pathway (Yang et al. 2020). Lu et al. found oridonin alleviated myocardial ischemia–reperfusion injury by preventing oxidative stress and NLRP3 inflammasome pathway (Lu et al. 2020). In addition, it was confirmed that oridonin exerted protective effects on LPS-induced acute lung injury and hind limb ischemia–reperfusion injury via Nrf2-independent anti-inflammatory and Nrf2-dependent antioxidative activities (Yang et al. 2019; Zhao et al. 2022). This study firstly proves that oridonin prevents atherosclerosis progression by suppressing NLRP3 inflammasome activation and promoting Nrf2 antioxidative stress.

We demonstrated that oridonin alleviated atherosclerosis in *ApoE*^*−/−*^ mice. In oridonin-treated atherosclerosis mice, histopathological data showed decreased macrophage infiltration, increased α-SMA positive area and collagen deposition, which indicated the regression of inflammation and an increase in the fibrous cap’s thickness, making the plaque more stable. Oridonin also reduced the area of lipid deposition and the size of atherosclerotic plaque. Oridonin administration resulted in a considerable reduction in the level of the pro-inflammatory cytokines IL-1β and IL-18. More importantly, oridonin not only inhibited the activation of NLRP3 and inflammatory response but also reduced ROS levels and oxidative stress. In addition, we explored the effect of oridonin on the lipid-handling capacity of macrophages. Here, we found that oridonin reduced lipid deposition in plaques by promoting lipid excretion and reducing lipid absorption.

**Fig. 7:**
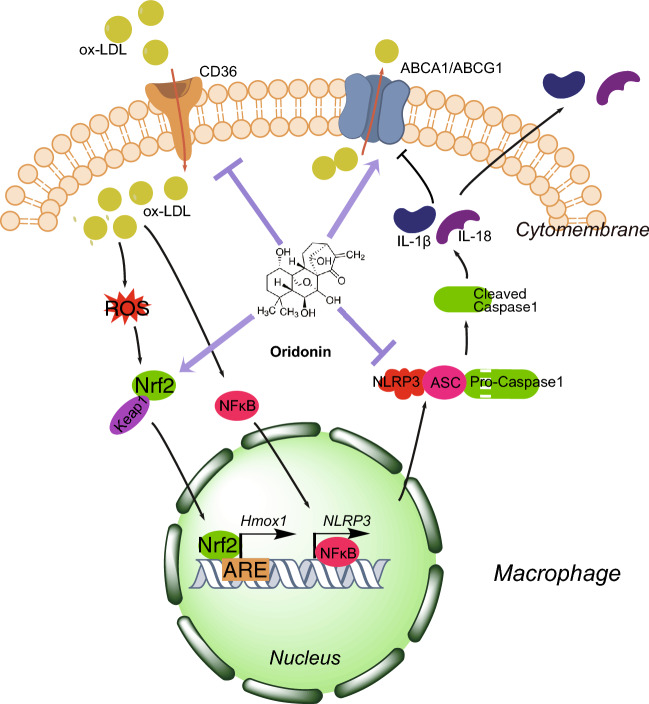
The mechanism of oridonin alleviating the progression of atherosclerosis. Oridonin attenuated atherosclerosis by inhibiting NLRP3-related inflammation and activating Nrf2-related anti-oxidative stress, resulting in suppressing lipid uptake and promoting lipid efflux

Our findings in this study are presented schematically in Fig. 7. In general, the evidence from this study suggests that oridonin has a protective effect on the development of atherosclerosis in *ApoE*^*−/−*^ mice. The mechanisms might be the inhibition of NLRP3 and the activation of Nrf2, which lead to lessened inflammation and oxidative stress in plaque, elevated lipid efflux and reduced lipid uptake in macrophages. Our study describes a meaningful and convincing pharmaceutical candidate for future clinical trials of atherosclerosis. It is necessary to further study the therapeutic mechanism and safe dose of oridonin in different atherosclerotic models.

Here are the proposed new messages. (1) Oridonin alleviated atherosclerosis, decreased lipid content and the infiltration of macrophages in plaque. (2) Oridonin reduced inflammation and oxidative stress in atherosclerosis. (3) Oridonin could inhibit NLRP3 inflammasome activation and activate Nrf2 pathway, resulting in suppressing lipid uptake and promoting lipid efflux.

## Data Availability

The data generated or analyzed during this study are included in this published article and its additional information files.
